# NIA Division of Behavioral and Social Research

**DOI:** 10.1093/geroni/igaf122.1556

**Published:** 2025-12-31

**Authors:** Elena Fazio

**Affiliations:** National Institute on Aging, Bethesda, Maryland, United States

## Abstract

Dr. Fazio will discuss research foci of the NIA Division of Behavioral and Social Research. Dr. Fazio will also be available for small group discussion.

